# A meta‐recovery framework: positioning the ‘New Recovery’ movement and other recovery approaches

**DOI:** 10.1111/jpm.12266

**Published:** 2016-01-21

**Authors:** G. Winship

**Affiliations:** ^1^ School of EducationUniversity of NottinghamJubilee CampusNottinghamNG2 5BYUK

## Introduction

This paper argues for a delineated explanation of the range of recovery approaches currently informing mental health interventions today. Four organizing domains of recovery are proposed: (1) Traditional Recovery; (2) Addictions Recovery; (3) New Recovery; and (4) Mutual Recovery. One of the challenges of providing mental health services efficiently is to consider which method of recovery is most suited to the needs of different service users. By comparing and organizing different recovery modalities, it is possible to consider the best fit between client and modality. For example, there is a necessary demarcation between clients who are amenable to recovery, and those who are harder to engage. We need to think about recovery in terms of the difference between ‘tender’ and ‘tough’ recovery approaches. A meta‐recovery framework is proposed here as a basis for continuing discussions about the different types of recovery operational in the field of mental health today.

## New Recovery

The present policy and practice momentum for recovery (DoH 2001, Expert Group on Mental Health Policy 2006, Scottish Executive 2006, Stickley & Wright 2011a, 2011b), which has emerged over the last two decades, has involved a different use of the term ‘recovery’ compared to the concept as it originated in self‐help movements such as Alcoholics Anonymous and Narcotics Anonymous, and then later in the field of substance misuse recovery. The semantics regarding the term ‘recovery’ itself has been subject to debate (Aston & Coffey 2012), and some colleagues have more lately talked about recovery with a big ‘R’ in order to denote the more recent use of the term (Edgley *et al*. 2012). However, in this paper, the term ‘New Recovery’ is proposed in order to delineate the more recent evolution of the concept of recovery compared to other more established and traditional approaches.

Although New Recovery has evolved from the field of psychiatric rehabilitation (Anthony 1993), over the last 10 years New Recovery ideology has seemingly placed itself increasingly at a distance from mainstream psychiatry (Bonney & Stickley 2008). There have been assertions that New Recovery is a radical departure, a paradigm shift on a par with the closure of asylums and the move to care in the community (Centre for Mental Health 2012). One of the most prominent developments in New Recovery has been the development of Recovery Colleges, and there are now 20‐plus Recovery Colleges in the UK offering a variety of ‘education courses’ ranging from short courses such as; ‘living with bi‐polar disorders’, to longer skills based and employment trainings. Tables 1 and 2 taken from a briefing document about the Recovery Colleges (Perkins *et al*. 2012) draw a distinction between a Recovery College‐based educative approach to recovery and a general overview of the process of therapy.

**Table 1:** From Perkins *et al*. (2012)

A therapeutic approach	An educational approach
Focuses on problems, deficits and dysfunctions;	Helps people recognize and make use of their talents and resources;
Strays beyond formal therapy sessions and becomes the over‐arching paradigm;	Assists people in exploring their possibilities and developing their skills;
Transforms all activities into therapies – work therapy, gardening therapy etc;	Supports people to achieve their goals and ambitions;
Problems are defined, and the type of therapy is chosen, by the professional ‘expert’;	Staff become coaches who help people find their own solutions;
Maintains the power imbalances and reinforces the belief that all expertise lies with the professionals.	Students choose their own courses, work out ways of making sense of (and finding meaning in) what has happened and become experts in managing their own lives.

**Table 2:** From Perkins *et al*. 2012

From day centre	To recovery college
Patient or client: ‘I am just a mental patient’	Student: ‘I am just the same as everyone else’
Therapist	Tutor
Referral	Registration
Professional assessment, care planning, clinical notes and review process	Co‐production of a personal learning plan, including learning support agreed by the student
Professionally facilitated groups	Education seminars, workshops and courses
Prescription: ‘This is the treatment you need’	Choice: ‘Which of these courses interest you?’
Referral to social groups	Making friends with fellow students
Discharge	Graduation
Segregation	Integration

The idea of shifting the role of patient to student is not without some controversy, and there might be some discussion about which descriptor is most empowering; however, positioning recovery as an education process is an idea that might have met with the approval of Peplau (1957):

If nurses focus on the learning possibilities and view psychiatric hospitals as special education institutions in which neglected learning – gaps in learning by experience in the past – can be rectified, this can be an important element in the patient's recovery. (Peplau 1957, p. 147)

Peplau points to the importance of learning from experience, and indeed becoming an expert by experience is central to the story of New Recovery and its ‘pioneers’. Pat Deegan is perhaps best known, an American who was diagnosed with schizophrenia as a teenager who recovered and then trained as a psychologist becoming an activist in disability rights (Deegan 1993, 1994). As a highly successful teacher, speaker, trainer, consultant and later as a business entrepreneur, Deegan began to attract followers in the United States, and then in the UK. Deegan argued that the goal of recovery was not to become normal, but rather to embrace the idea of becoming more fully human, that is to say, becoming a person rather than being seen as a collection of psychiatric symptoms (Deegan 1996). As Deegan's work came to prominence in the UK, it offered an anchor for others who had become experts by experience, for example Rufus May, best known for his part in the documentary ‘The Doctor who hears voices’, which was first screened by Channel 4 in 2008 (Regan 2008), who had recovered from psychosis and went on to qualify as a psychologist. Rufus was part of a new wave of experts by experience in the UK who defined the New Recovery movement shaping the field of mental health service provision, education and research (Simpson *et al*. 2008).

## A meta‐recovery framework: comparing recovery approaches – where are the overlaps?

So, how does New Recovery stack up against other recovery facing approaches? We might say that key ideas such as kindness, hope and respect, are concepts long since embedded in psychiatry since Samuel Tuke's (1813) influence at the York Retreat (Roberts & Boardman 2013); and it is arguable that many key ideas of New Recovery, such as increasing peer involvement, challenging the role of the expert, flattened hierarchies and patient empowerment, have been centrifugal to the philosophy of progressive practice in social psychiatry since the 1940s (Rapoport 1960, Winship 1996, Campling & Haigh 1999). For the purpose of debate here, Table 3 offers a provisional overview of what we might think of as the four major domains of recovery, with a brief comparative summary of characteristics of approach and the suitability of clients to each approach. The table is not intended to be an exhaustive review, but rather a brief synopsis as the basis for discussion. Four approaches have been delineated, but in practice it is likely that many service users will experience an overlap of the different approaches.

**Table 3:** A meta‐recovery framework

**Traditional Recovery** **When:** 1880s–present **Brief**: Based on models of rehabilitation for hospitalized patients (Benbow & Bowers 1998). Social psychiatry (Jones 1968), community psychiatry, group therapy, therapeutic communities (Dietrich 1976, Hinshelwood & Manning 1979), psychosocial interventions **Theoretical orientation**: Clinical recovery: diminution of symptoms (Onken *et al*. 2007, Harvey & Bellack 2009), industrial therapy units (Wells 2006), biological model, often accompanied by pharmacological intervention, although some non‐pharmaceutical approaches (e.g. Soteria, Arbours). A range of psychological therapies deployed. Clients' suitability: including involuntary and detained, patients suffering more acute or severe episodes requiring more intensive interventions, hospitalization, residential treatment or day hospitals, secure treatments, therapeutic prisons.	**Addictions Recovery** **When:** 1930s–present **Brief**: Large international network of addictions recovery approaches, often using Therapeutic Community principles. Peer self‐help group movement committed to recovery and sobriety. Later Narcotics Anonymous. Most addiction recovery programmes emphasize the importance of staged steps towards recovery, and the importance of peer relationships, prosocial encounters in therapy which addresses the antisocial compulsions of substance misuse. Many addiction recovery programmes employ people ‘in recovery’ as therapists, experts by experience. **Theoretical orientation**: 12‐Step, Milieu Therapy, Minnesota Model, Concept House approach, TCs, relapse prevention, replacement prescribing (route to detoxification), correctional institutions (US). Clients: people suffering from drug and alcohol problems, also other compulsions such as eating disorders or gambling.
**New Recovery** **When:** 1990s–present **Brief:** National Health Service, Psychiatry, Recovery Colleges, non‐residential, private entrepreneurships (especially US). **Theoretical orientation:** Education focused, anti‐therapy, Recovery Colleges, consumer led, entrepreneurial, co‐construction, with a focus on hope‐inspiring relationships, both with peers and staff (Slade 2009). Recovery features social inclusion, clients are ‘valued as human beings’ and where staff offer belief in the person's ability and potential. Changing practice including risk assessment and redefining user involvement (Boardman & Shepherd 2009). Socially focused‐based approaches that included strategies for facilitating a befriending, health information, social skill and life skills and so forth, with a strong Rogerian underpinning (Repper & Perkins 2003). Client suitability: People who are able to voluntarily engage with recovery and educative approaches, clients with longer term conditions that require less intensive intervention.	**Mutual Recovery** **When:** 2011–present **Brief:** Initially Arts & Humanities Research Council Funded Research (1.5million to establish and trial research looking across a range of arts interventions) focusing on third sector, independent, non‐residential services including arts centres, galleries, libraries. **Theoretical orientation**: Artists take the lead in programme design and delivery. Current programmes include; music, clay sculpting and creative writing, photography, drumming, Capoeira, music, digital storytelling, yoga, reading circles, performance arts workshops (e.g. comedy, poetry) (Crawford *et al*. 2013). Client suitability: People who are able to voluntarily engage with recovery and interested in arts‐based approaches, clients with longer term conditions requiring less intensive intervention.

Traditional Recovery has been built on the challenge of rehabilitation and has been closely allied to the medical model with an emphasis on biological explanations of illness, and biological solutions such as pharmaceutical intervention. However, it also true that Traditional Recovery has progressed towards a more humanized model of psychiatry (Pilgrim & Rogers 1993), shaped by the emergence of social psychiatry and therapeutic community (TC) informed practices (Jones 1968). Therapeutic Community practice foreshadows New Recovery insofar as TCs have historically redefined the terms of engagement between professional and service users by seeking to where flatten hierarchies and deploying the principles of democratic user empowerment in shaping the treatment milieu (Winship 1996, Yates 2011). Some TCs have more lately adapted and articulated their work in terms of the principles of New Recovery, for instance the Haven Project in Colchester (Castillo 2013) now talks about a concept they call *transitional recovery* (Castillo 2003, 2009, 2013), where the approach recognizes stages in the recovery journey making explicit that recovery is not an end point but rather a series of steps towards well‐being.

Addiction Recovery has also been well established since the 1930s and evolution of Alcoholics Anonymous, a movement led by Bill Wilson and Dr Bob Smith. From the late 1960s, there has been a growing number of detoxification and recovery programmes, largely in the National Health Service in the UK (Winship 2014) and correctional approaches in the United States (De Leon 2000) and commonly informed by therapeutic community principles around the globe (Abdollahnejad 2008, Paget *et al*. 2008). On the surface, there might be value in bridging Addiction Recovery methods and the principles of ‘New Recovery’ to the mutual benefit of both approaches (Yates & Malloch 2010). For example, by looking across New Recovery and models of Addiction Recovery such as a 12‐step programme, one could map ‘relapse signatures’ in the journey to recovery (Marland *et al*. 2011), especially where recovery involves overcoming a co‐occurring substance misuse problem (Miller *et al*. 2005).

The newest recovery paradigm in the quadrant has emerged from an emphasis on applying the arts in the service of what has become known as ‘Mutual Recovery’ which focuses on recovery facilitated by artists rather than mental health practitioners or educationalists (Spandler *et al*. 2007, Crawford *et al*. 2013). Mutual Recovery is an approach that diminishes the role of health expert and emerges on the crest of a ‘new wave of mutuality’ with renewed interest in cooperation in psychiatry (Murray 2012). The new raft of Mutual Recovery programmes have included workshops and intervention that have covered a wide range of arts such as clay sculpting, storytelling, stand‐up comedy, photo therapy and have been supported by the Arts & Humanities Research Council. The Mutual Recovery method has sort to engage with service users as well as family members, informal carers artists and professionals providing a therapeutic environment which is conducive to recovery (Argyle 2015). The Mutual Recovery approach aims to build egalitarian, appreciative connected communities which are geared towards recovery for clients, carers and professionals alike (Crawford *et al*. 2013).

Perhaps one of the overarching overlaps between all of the different recovery approaches is the fact that in each area, ideas have been informed by the vision leaders who have been described as experts by experience or wounded healers. The concept of ‘wounded healer’, that is to say, someone who has experience of the distress and illness that they then seek to help in others, has a lineage from Jung (1963) onwards informing the progress of mental health professions, from psychology to social work and especially in counselling and psychotherapy (Fussell & Bonney 1990, Nouwen 1990, Black *et al*. 1993, Elliott & Guy 1993, Sedgwick 1994, Murphy & Halgin 1995, Cain 2000, Jackson 2001, Olson & Royse 2006, Barnett 2007, Sussman 2007, Ivey & Partington 2012, Adams 2014). In the history of TCs, there have been a number of examples of wounded healers. For example in the late 1970s, the Charles Hood Unit TC at the Bethlem and Maudsely was closed as a result of the breakdown of the lead psychiatrist Bob Hobson (cf: Hinshelwood & Manning 1979). The event was seen as seen as something as a matter of chagrin rather than as a case where the reality is that professionals might too be prone to mental health vulnerabilities. R.D. Laing's personal battles with depression and alcohol dependence were also factors which informed the TC experiment at Kingsley Hall, but were likewise considered as matters of professional embarrassment rather than experiences which were seen as informative (Laing 1994). The same story might be said of the psychiatrist Julian Goodburn, who led the Paddington Day hospital therapeutic community in the late 1970s before his highly publicized breakdown (Spandler 2006). More lately, training in psychotherapy and therapeutic community practice commonly involves trainees undergoing a period of personal therapy, which might entrain the idea that practitioners need to recognize their own mental health vulnerabilities as part and parcel of training and practice governance.

## ‘Tender’ and ‘tough’ recovery – the difference between New Recovery and other recovery models

Having looked at some areas of overlap in relation recovery paradigms, what might be considered as points of departure? New Recovery begins with a pre‐supposition of client cooperation or at least some level of willingness on the part of the client to engage in a process of recovery, and in terms of the Recovery College approach, a willingness to become a student in a process of education. However, in acute psychiatry, the process of recovery often begins with the client in a state of retreat, where there is reluctance on the part of the client to engage with recovery, where the initial encounter is characterized by conflict rather than cooperation. Not all clients engage with services with the motivation to recover. Instead, clients present with complex demands. Therapeutic Community methods have been historically whittled from residential experiences of working with people who suffer with psychotic states, or anti‐social disorders where the first challenge is that of reaching a point of mutuality and cooperation.

The necessary capacity to engage in the dynamics of conflict might be characteristic of what has become known in the field of addictions recovery as ‘tough love’; a term that has been used from the 1960s onwards and has since become parlance in the approach of concept houses and other TC‐minded services for addiction and eating disorder recovery. We might think of ‘tough recovery’ in contrast to a more ‘tender’ approach of New Recovery. Therapeutic communities have sometimes had the reputation of being harsh and challenging places to be. There may be something in the lineage of TCs dating back to war time experiences with the TC method having its roots in the army as a treatment method of recovery that dates back to the 1940s in the UK with shell shocked soldiers casualties from the Second World War (Bion 1946, Trist 1985, Harrison 2000, Winship *et al*. 2009). Clients can initially find the experience of being in a TC structured where the forthright inclination towards social inclusion can feel like a confrontation.

The reputation of TCs being rather tough places to recover might not be unreasonably cast. The idea of tough recovery is best encapsulated in the idea of ‘reality confrontation’, a phrase coined by Rapoport (1960) based on his observation of work at the Henderson Hospital. Although TCs today have many other quintessential elements such as ‘containment’ and ‘agency’ (Haigh 2007), the idea of reality confrontation in TCs has never been entirely dispatched. Reality confrontation is associated with a sort of encounter group culture where emotional confrontation was thought to be necessary to the journey of recovery. Reality Confrontation is seen as an ingredient in helping a client begin to cope with the demands of everyday life, taking the form of the client participating in the activities of daily living such as cooking or cleaning. Critics of the TC approach might argue that a vulnerable client needs protecting from reality. Though it should be stressed that for TC clients, reality confrontation is not always a delivered as a big dose so to speak, rather it is more like reality is experienced in small everyday doses. For example, a client in a TC might take on a new role initially such as helping with cooking or cleaning, and then later take on a more difficult role such as chairing meetings. Reality Confrontation in TCs is more like Winnicott's (1965) idea that good enough maturational environments are characterized by life in small doses. Therapeutic community clients progress through their journey to recovery in small steps, and they only assume bigger roles of responsibility as time progresses. In other words, reality confrontation is a stepped process with new clients having much less responsibility, with clients longer in treatment assuming more roles of responsibility.

In trying to think about phases of recovery, and the suitability of models of recovery for particular client needs, we might consider Tough Recovery as an initial stage of recovery which pre‐dates the client's readiness to become a student of New Recovery. That is to say, we need to think about the transition from acute stages of illness to recovery interventions when the client has less severe suffering. Ideally, all people on the pathway to recovery should have the opportunity for learning and education, much as Peplau envisages the hospital and health‐care system as an educative endeavour. The reality however, is that many clients need to go through stages of recovery where their acute needs dictate a more active role on the part of professionals before they are ready for more tender phases of recovery.

## The politics of recovery

One of the distinguishing features of the New Recovery approach is that it has been carved out of the more individualistic tendencies of social entrepreneurism. Deegan's New Recovery work in the United States is largely a private industry and there can be no doubt that the New Recovery method in the UK has sought to diminish the role of state intervention, replacing professional input with a workforce that aims is to reduce costs.

The concept of self‐help and self‐organization replaces the role of professionals and institutions and the approach emphasizes resilience rather than vulnerability (Friedli 2009). The inclination is therefore positively focused on the resources people have at their disposal, which has been referred to as an assets‐based approach (Burns 2011) where the development of ‘recovery capital’, that is to say, the array of social, psychological and cultural networks beyond professional inputs, is considered to be requisite to sustaining the journey towards recovery (Best & Laudet 2010, Yates 2014). We might think of New Recovery, as with other recovery approaches which seek to diminish professional input with lean costs, as a paradigm fit for austerity. On a political spectrum, New Recovery can be situated as a liberalist approach (Edgley *et al*. 2012), compared to TCs, which might be considered as deriving from more left democratic leanings framed by collectivism rather than individualism (Winship 2004, 2013).

It might be useful to consider the present enthusiasm for New Recovery against the backdrop of the other recovery movements which have shared similar ambitions. For example, if we look back at the history of TCs we can see how they went from movement to method and then later to a set of maxims with the onset of monitoring, measurement and critique. The journey from movement to method to maxim for the TC movement took place across a period of 70 years or more, and along the way there were many books, research, the establishment of a journal and a dedicated archive; the Planned Environment and Therapy Trust. But by comparison, the New Recovery approach has gone from being a movement (in the 1990s) to a method (from 2001 onwards in the UK with the publication of policy documents) and now embedded as a set of maxims (2008 onwards). It has been a journey that has been less than 20 years. We might think of New Recovery as a stellar rise of a new paradigm or as a sprint of opportunism built on the stilts of charismatic leadership. The TC movement has long since understood the precariousness of charismatic leaders (Manning 1989, Campling & Haigh 1999), charismatic ideas are preferred to charismatic leaders, with proof of concept as the best footing for methodological progression (Davies & Campling 2003).

Family trees are essential to root one in the present and to a set tenure for future development. The New Recovery Movement, in the process of inventing itself without recourse to the lineage of allied and influencing trajectories, might well have inadvertently written itself into an ideological vacuum. New Recovery might do well to reflect on the way in which other branches of recovery have sustained their practices long after the first wave of leaders became memories.
